# Human corneal cell culture models for drug toxicity studies

**DOI:** 10.1007/s13346-016-0330-y

**Published:** 2016-09-09

**Authors:** Seppo Rönkkö, Kati-Sisko Vellonen, Kristiina Järvinen, Elisa Toropainen, Arto Urtti

**Affiliations:** 1 School of Pharmacy, Faculty of Health Sciences, University of Eastern Finland, P.O.Box 1627, 70211 Kuopio, Finland; 2 Centre for Drug Research, Division of Pharmaceutical Biosciences, Faculty of Pharmacy, University of Helsinki, P.O. Box 56, 00014 Helsinki, Finland

**Keywords:** Ocular toxicity, Corneal cell culture, ADME prediction, In vitro model, Ocular bioavailability

## Abstract

In vivo toxicity and absorption studies of topical ocular drugs are problematic, because these studies involve invasive tissue sampling and toxic effects in animal models. Therefore, different human corneal models ranging from simple monolayer cultures to three-dimensional models have been developed for toxicological prediction with in vitro models. Each system has its own set of advantages and disadvantages. Use of non-corneal cells, inadequate characterization of gene-expression profiles, and accumulation of genomic aberrations in human corneal models are typical drawbacks that decrease their reliability and predictive power. In the future, further improvements are needed for verifying comparable expression profiles and cellular properties of human corneal models with their in vivo counterparts. A rapidly expanding stem cell technology combined with tissue engineering may give future opportunities to develop new tools in drug toxicity studies. One approach may be the production of artificial miniature corneas. In addition, there is also a need to use large-scale profiling approaches such as genomics, transcriptomics, proteomics, and metabolomics for understanding of the ocular toxicity.

## Introduction

Cornea is an effective absorption barrier for topically applied ocular drugs, but at the same time it is the most significant route for drug permeation to the anterior chamber [1]. Therefore, isolated animal corneas and cultured corneal epithelia have been used to study drug permeability in the cornea [2–4]. In vivo biodistribution studies require sacrification of at least 20 animals (e.g., 5 time points, 4 eyes/point, 2 drugs or formulations compared), typically rabbits, because non-invasive sampling is not possible and many animals must be killed at each time point in order to generate the concentration curves [5–7]. The role of corneal cell models in permeability testing has been reviewed previously [8, 9].

As a drug permeation route, the corneal cells are exposed to the potential toxic effects of the applied drugs. Traditionally, the corneal and other ocular toxicity has been studied in animal experiments, but such experiments (e.g., Draize test) have been widely criticized for ethical reasons. In Draize test, the test substances are instilled into the lower conjunctival sac of an albino rabbit [10]. The conclusions are drawn based on the observed changes in the anterior segment of the eye. The possible changes include corneal opacification, conjunctival redness, iritis, edema, and lacrimal discharge. Evaluation of the results is subjective and dependent on the person, who is examining the eyes. The rabbit model has also been criticized for the differences in physiology, anatomy, and morphology between human and rabbit eyes. In addition, the test is not truly quantitative, and the test may cause pain and/or discomfort to the animals.

Ex vivo animal-based models have also been used in ocular toxicity assessment. These methods include isolated tissues (cornea) and organs (whole eye) [11, 12]. Corneal opacity and permeability (BCOP) assays are based on intact corneas isolated from bovine tissues, whereas the isolated chicken eye (ICE) test is used to follow toxic reactions after applying the test substance to the cornea of whole chicken eye. These methods allow measuring of the cytotoxic effects such as changes in opacity, fluorescein retention or permeation, tissue swelling, and other macroscopic changes. Although normal physiological and biochemical properties are present, these models are suitable only for short-term (a few hours) assessment of toxicity. However, assessment of toxicity with animal tissues may not represent the conditions in the human eye.

Recently, ocular toxicity tests have been increasingly performed with in vitro methods [13]. The authorities have encouraged researchers to develop in vitro studies, for example, the European legislation (Directive 63/2010/EU) is based on replacement, reduction, and refinement of animal experiments. Furthermore, in 2013, the European Union banned animal testing for cosmetics (Cosmetics Directive 76/768/EEC). Even though the directives allow medical research with animals, the recommendations and legislation will probably shift toward the alternative methods.

In recent years, a variety of human corneal cell models in vitro have been developed [4, 14–18]. In the simplest model, human corneal epithelial primary or immortalized cells are grown in conventional cell culture wells. The more sophisticated systems are based on the culture of the cells on extracellular matrix-coated filters allowing generation of polarized three-dimensional corneal models. Furthermore, cell culture models that mimic the entire human cornea have been developed. This review gives an overview to the properties of the corneal cell culture models used in ocular toxicity testing.

## Human corneal cell models

Human corneal cell culture models have been developed for studies of corneal permeation and barrier studies [4, 15–17], toxicity testing [19–23], and ocular transport studies [24]. These models use primary and immortalized cell cultures and different 3D corneal equivalents as well.

### Microscopic anatomy of human cornea

**Fig. 1:**
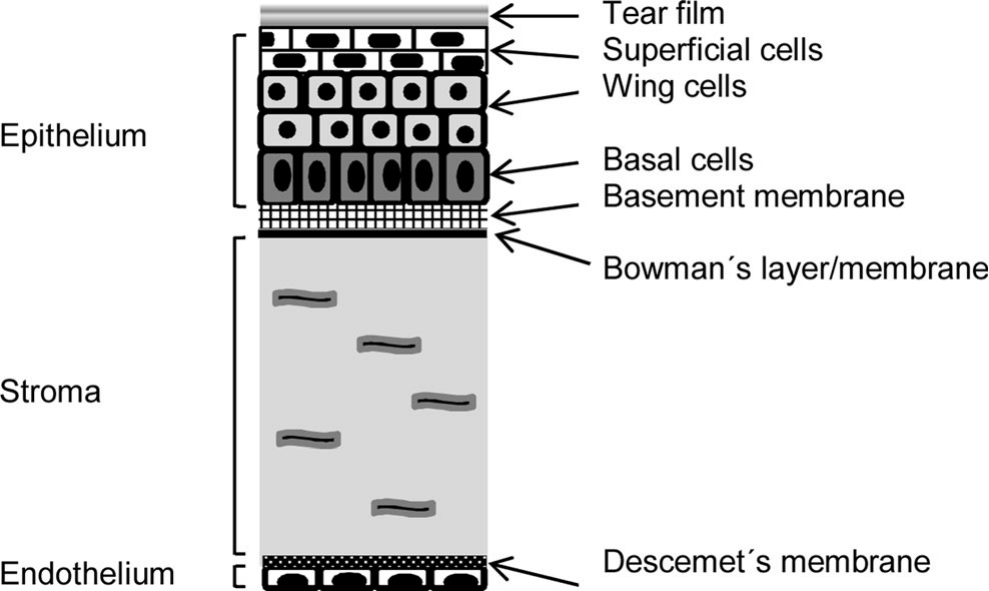
Schematic representation of the different corneal layers

The cornea is an avascular and transparent tissue between tear film and anterior chamber. The tear film keeps the cornea moist and protects the eye against infections [25]. The cornea is a multilayered tissue consisting of epithelium, basement membrane, Bowman’s layer, stroma, Descemet’s membrane, and endothelium (Fig. 1). The epithelium has five to six cell layers, with a total thickness of about 50 μm. The two most anterior cell layers of the corneal epithelium are flattened and contain tight junctions. Below these superficial cells are the multilayered wing cells and one layer of mitotically active columnar basal cells [18, 25]. The basement membrane (40–60 nm) contains collagen IV, laminin, and fibronectin, and it plays an important role in the maintenance of the corneal epithelium [18]. Bowman’s layer/membrane (8–12 μm) is acellular consisting of randomly arranged collagen fibers. The corneal stroma (500 μm) consists of 2-μm-thick flattened collagenous lamellae. The collagen fibers are mainly of hydrated type I collagen with interspersed glycosaminoglycans (GAG) and some type III, V, and VI collagen [25]. Between the lamellae lie flattened keratocytes which are located throughout the stroma with a density of 20,000–24,000 cells/mm^2^ in humans. Gap junctions connect keratocytes to their neighboring cells to maintain the structure and transparency of the stroma. Keratocytes produce extracellular matrix proteins and can synthetize collagen for tissue repair [26–28]. Acellular Descemet’s membrane (7 μm thick) beneath the stroma is composed of collagen fibrils. The endothelium is a single layer of hexagonal-shaped cells with a total thickness of about 5 μm. It covers the innermost surface of the cornea, but it does not resist permeability of drugs to the aqueous humor. Recently, Dua et al. [29] introduced another layer in the human cornea. This acellular layer of 10 μm exists between Descemet’s membrane and stroma. However, so far, no other researcher group has confirmed their finding.

### Human corneal epithelial primary cell cultures

**Fig. 2:**
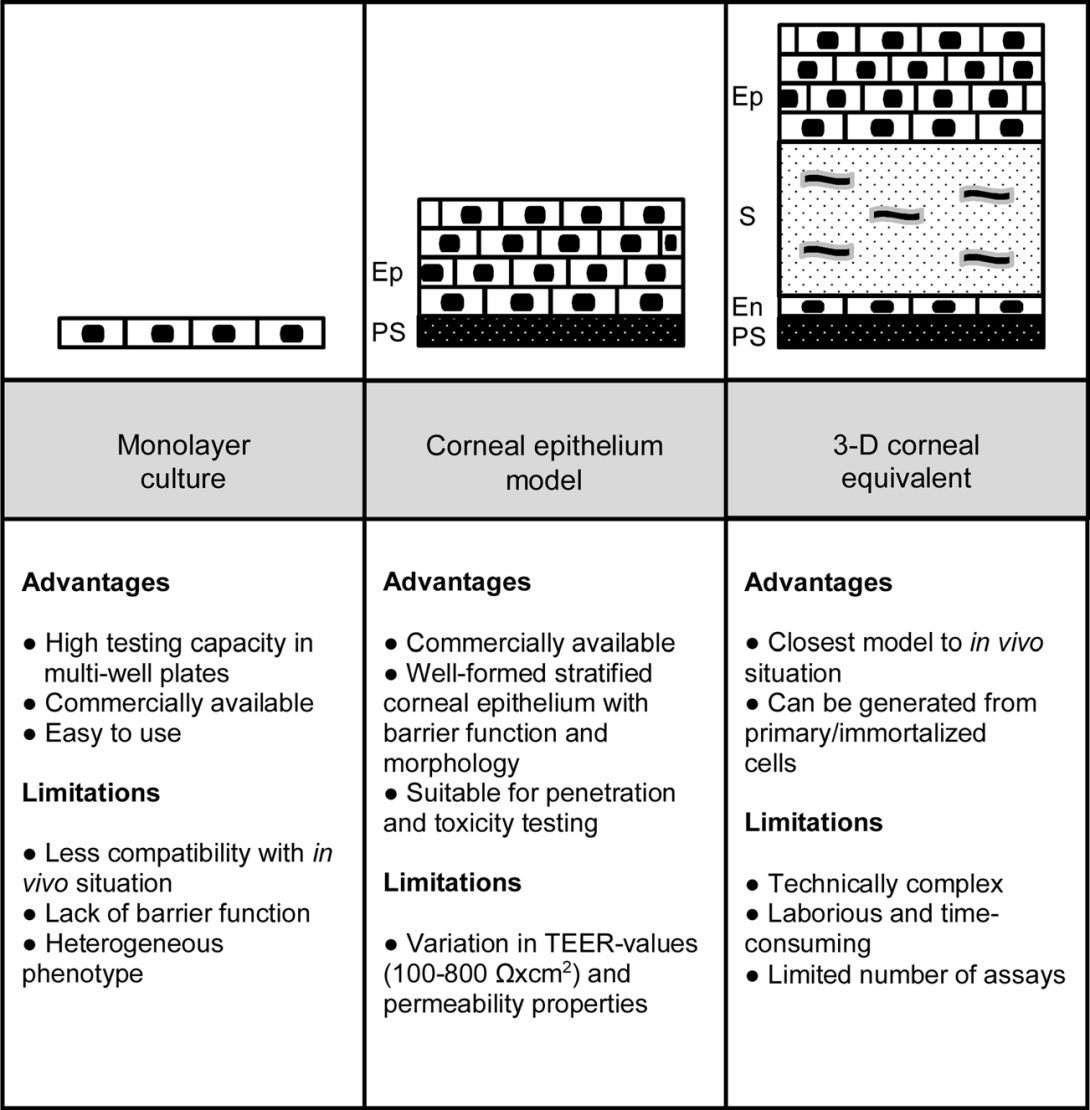
Schematic presentation of various human corneal culture models with their advantages and limitations. Abbreviations are endothelial cells (*En*), epithelial cells (*Ep*), permeable support (*PS*), and stromal cells (*S*)

Human primary corneal epithelial cells [21, 30–37] have been used in a wide range of basic ocular studies involving cell attachment, cellular uptake, apoptosis, toxicity, and effects of growth factors on epithelial cell proliferation and differentiation. The primary cultures of the cells are not modified thereby representing native corneal epithelial cells. Furthermore, the primary cultures are easy to use when compared to 3D corneal epithelial models and corneal equivalents (Fig. 2). Although human primary corneal epithelial cells are commercially available from many suppliers, they are not optimal for in vitro use due to their short life span up to about ten passages [38], but the phenotype of the primary cells has often better match with the in vivo tissue as compared to the modified cell lines. Primary human corneal epithelial cells are useful in toxicity studies using various end-points (“Toxicity tests with corneal cell culture models” section). They can be used in normal cell culture wells or as components to generate more complex cell models.

### Immortalized human corneal epithelial cell lines

The life span of corneal primary cells can be extended using viral immortalization techniques (e.g., recombinant SV-40-adenovirus, recombinant retrovirus). Immortalized human corneal epithelial cell lines, such as the HCE-T [39], CEP1 or CEP1-17-CL4 [40], 10.014 pRSV-T [38], and HPV16-E6/E7 [41], have been generated with viral genes. Sometimes, human corneal primary cells undergo spontaneous immortalization [42], but immortal cells may exhibit altered growth, tumorigenicity, and abnormal levels of proteases and cell surface markers. Furthermore, immortalized cell lines have chromosomal abnormalities and may contain heterogeneous cell populations [43]. Corneal cell lines with extended life-span offer several advantages compared to the primary cells: unlimited renewal of the cells, more reproducible experiments, and easier genetic manipulation. These cells may, however, undergo different responses to the toxic chemical exposure as their gene expression profile can differ from the normal corneal epithelium; for example, the efflux protein expression is increased compared to the normal corneal epithelium and this may reduce the cellular exposure to the external chemicals [24]. This is also a common mechanism for cancer cells to acquire resistance to the cytotoxic agents.

The Statens Seruminstitut rabbit corneal (SIRC) cells have also been used as a model of human corneal epithelium [17, 18], but in fact the SIRC cells have a fibroblast phenotype [44].

Corneal cell lines are useful in toxicity studies using various end-points, and their performance in normal cell culture wells without differentiation was successfully correlated with the in vivo tolerance of some drugs, excipients, and formulations [22]. Only human cell lines should be used. Since primary corneal epithelial cells and cell lines are commercially available, there is no valid reason to use SIRC cells in ocular toxicity evaluations.

### Corneal epithelial cell models on filters

When corneal epithelial cells are cultured on filters (e.g., Transwell), they will polarize and form a barrier that resembles the normal corneal epithelium [4] enabling investigations on chemical impact on the barrier function (Fig. 2). The differentiation process and quality of the barrier depends on the filter material and its coating [4]. These culture models can be based solely on the corneal epithelial cells (primary or immortalized) or alternatively on three-dimensional models with corneal epithelial cells and feeder cells (e.g., human-derived epidermal keratinocytes). Typically, the corneal epithelium is formed after air-lift, i.e., culture on air-liquid interface [4, 18, 45–47]. Usually, the corneal epithelial models contain 3–10 epithelial cell layers, but not the stromal or endothelial layers. In toxicity studies, these models inform only about the toxic responses that are corneal epithelium borne.

Generation of a corneal epithelial model on a filter is a long process, since differentiation may take several weeks and, in the case of secondary cell lines (like HCE), the filter grown epithelium does not always differentiate properly. This model has limited advantage over a simple culture of the non-differentiated cells in the culture wells. For example, the influence of the chemicals on barrier function is the mechanism that can be studied on filter grown cells, but not in the normal wells. Otherwise, there is no proof that this more complex cell culture set-up would have an advantage over simple culture in toxicity predictions.

### Three-dimensional cornea equivalents

Three-dimensional corneal tissue equivalents are usually generated using three corneal cell types (epithelial, stromal, and endothelial cells). The three-dimensional cornea equivalent is built step-by-step (Fig. 2). The main advantage of these models is the complete corneal architecture that resembles the in vivo situation.

The first three-dimensional corneal equivalent was reported in 1999 [48]. This model was consisted of epithelium, stroma, and endothelium that were based on the immortalized human corneal cells. This construct mimicked human cornea in terms of morphology, expression of some markers, transparency, and ion and fluid transport. Later, another corneal equivalent model was introduced as a tool for ocular irritation tests [49, 50]. The culture model was based on SV40-immortalized human corneal epithelial cells, human corneal keratocytes, and human corneal endothelial cells.

The keratocytes play an important role in 3D cell culture models, since they are located in the stroma between the epithelial and endothelial cell layers. The keratocytes maintain the structure and transparency of the stroma. The culture media for keratocytes contain many substances, such as FBS (~10 %), ascorbic acid, insulin, transferrin, selenium, and recombinant growth factors (epidermal growth factor, basic fibroblast growth factor, platelet-derived growth factor, and leukemia-inhibitory factor) [28, 51, 52]. Low-level nutrition may disturb the normal cell metabolism leading to abnormal phenotypic changes also in the presence of serum [28, 52]. At low serum level (equal or below 2 %), the cells retain their keratocyte phenotype.

Three-dimensional cornea equivalents are in principle optimal models for corneal toxicity studies, because they mimic the entire tissue, including the epithelium, stroma, and endothelium. Even though all the layers are present, the biochemical pathways and toxicological reactions may still differ from the normal cornea. Unfortunately, the toxic responses of the 3D cornea equivalents have not been rigorously studied or compared with the normal cornea. Therefore, the true benefits of these complex models in comparison with the simpler models are still open. One potential advantage is related to the studies with erosive compounds, since monitoring of corneal erosion requires intact corneal tissue.

### Corneal endothelial cell models

The corneal endothelium has an important role in the maintenance of the health, but human corneal endothelial cells do not normally undergo mitosis in vivo because these cells have arrested into the G_1_-phase of the cell cycle [53]. Therefore, the cell density gradually decreases with age [54], and these cells recover poorly from the damages. Therefore, the corneal endothelium is an important cell type from a toxicological point of view.

Isolated human corneal endothelial cells are able to proliferate only for a few passages under certain in vitro conditions. Increased cell proliferation is achieved using insulin, growth factors (nerve growth factor, epithelial growth factor, and basic fibroblast growth factor), bovine pituitary extract, ascorbic acid, serum, and mitogens [55–57]. Chondroitin sulfate, one of the GAGs in the human cornea, is also used in the media [57], since it acts as a scaffold material of extracellular matrix (ECM) template that is needed for proper cell growth and organization [58]. Kim et al. [57] compared four different media for corneal endothelial cell culture. Traditional human corneal endothelial cell medium with FBS, epidermal growth factor, basic fibroblast growth factor, and chondroitin sulfate was the best medium in terms of stem cell-associated protein expression, cell proliferation, and migration. However, the cells become elongated and more fibroblast-like than in the stem cell media that was more appropriate for maintaining the correct cell shape and functionality. Fibronectin-collagen, laminin, and collagen type I are often used as a substrata for the endothelial cells [57, 59–61]. These compounds have influence on cell adhesion, proliferation, and migration.

In that case, donor-to-donor variability and culture medium composition have great impact on growth rate, proliferation capacity, and morphology of the cells [56]. Proliferation activity of human primary corneal endothelial cells depends on age of donors [62]. Age-related nuclear oxidative DNA damage decreasing the proliferative capacity of the cells [63]. Sometimes, corneal endothelial cell preparation may be contaminated by proliferative stromal fibroblasts leading to batch-to-batch variation in the primary cell cultures. To increase the life span of these cells, immortalized human corneal endothelial cell lines have been established by telomerase engineering [64]. These immortalized human corneal endothelial cells had endothelial hexagonal morphology, and they were able to proliferate more than 70 passages without signs of senescence.

Corneal endothelial cells are a useful model when endothelial toxicity is specifically studied, but ocular irritation is not necessarily related to the endothelium that is located deeper in the corneal tissue.

### Corneal stem cells

Corneal integrity and function depend on the self-renewing properties of the corneal epithelial cells. Fully differentiated, superficial cells of the corneal epithelium are continuously shed from the ocular surface so that the complete turnover of the corneal epithelium takes place in 7 days. During that time, basal cells migrate upward from the basal layer, and differentiate into wing cells and superficial cells [65]. Corneal stem cells serve as an important source of new basal cells.

Different types of stem cells exist in the corneal epithelium, stroma, and endothelium [66–69]. For epithelial stem cells, the main hypothesis suggests that the epithelial stem cells are located in the corneal limbus that is a transition zone between the corneal and conjunctival epithelia [66, 67, 70, 71]. Experimental evidence suggests that a unique microenvironment in the limbal palisades of Vogt may be responsible for the maintenance and function of corneal stem cells [72, 73]. An alternative hypothesis proposes that stem cells are located in the basal layer of the corneal epithelium [74], but most corneal stem cell research has been focused on limbal stem cells. There is also some experimental evidence supporting the existence of stem cells in the corneal stroma and endothelium [69, 75, 76]. As far as we know, there are no reports on the utilization of human corneal stem cells in toxicological models.

### Tissue engineering applications for corneal cells

Tissue engineering approaches utilize cellular engineering and biomaterials to generate artificial tissues for transplantation. Similar approaches can be used to develop cell models for drug and chemical testing.

**Fig. 3:**
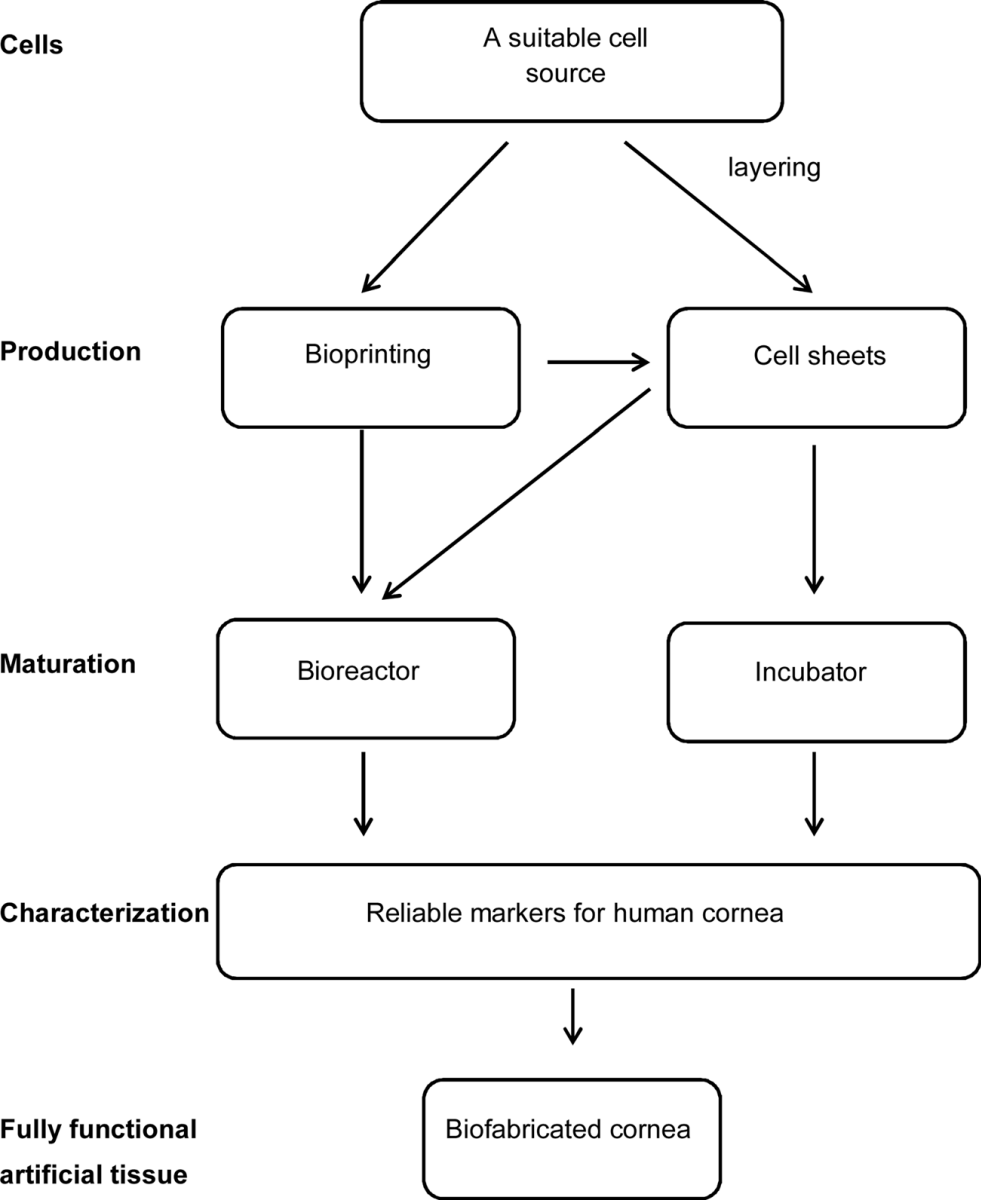
Basic steps in tissue engineering techniques for 3D biofabricated cornea formation. Isolated or cultured cells can be printed in the desired format and then further grown as layers or cultured in bioreactor for characterization and use in toxicological testing

In general, suitable biochemical factors of the medium must be combined with a biomaterial scaffold in which the cells are grown [77] (Fig. 3). Three-dimensional cell constructs should mimic the target tissue to achieve the desired functionality. In the human eye, cell-based and scaffold-based corneal engineering have been used in corneal transplantation [78]. For example, grafts of autologous limbal stem cells on fibrin [79] or on amniotic membrane [80, 81] have been used to restore the damaged corneal surface. In animal models, collagen, amniotic membrane with gelatin, and poly(*N*-isopropylacrylamide) (PNIPAAm) have been used for tissue engineering of corneal endothelium [82]. Thermo-sensitive PNIPAAm is a promising polymer [83], since a temperature change from 37 to 20 °C modifies its structure thereby releasing intact cell sheets with extracellular matrix from the PNIPAAm support [84]. Tissue structure is not damaged, because proteolytic enzymes are not needed [85]. This technique may open new possibilities to develop corneal epithelia and corneal equivalents by harvesting and layering from bottom to top human corneal endothelial, stromal, and epithelial cell sheets. In multi-layered construct, distinct cell sheets may spontaneously integrate resulting in a corneal in vivo tissue substitute.

Tissue engineering methods have also limitations. For example, synthetic matrix materials may have unfavorable properties, such as sub-optimal mechanical properties and inadequate support of cell growth [86]. The cell source is the greatest uncertain feature in corneal tissue engineering. Quality of limbal stem cells and primary cells (epithelial, endothelial) depends on the donor tissue leading to variations in the proliferation, cell density, and phenotype of resulting cells in the culture [87, 88].

**Fig. 4:**
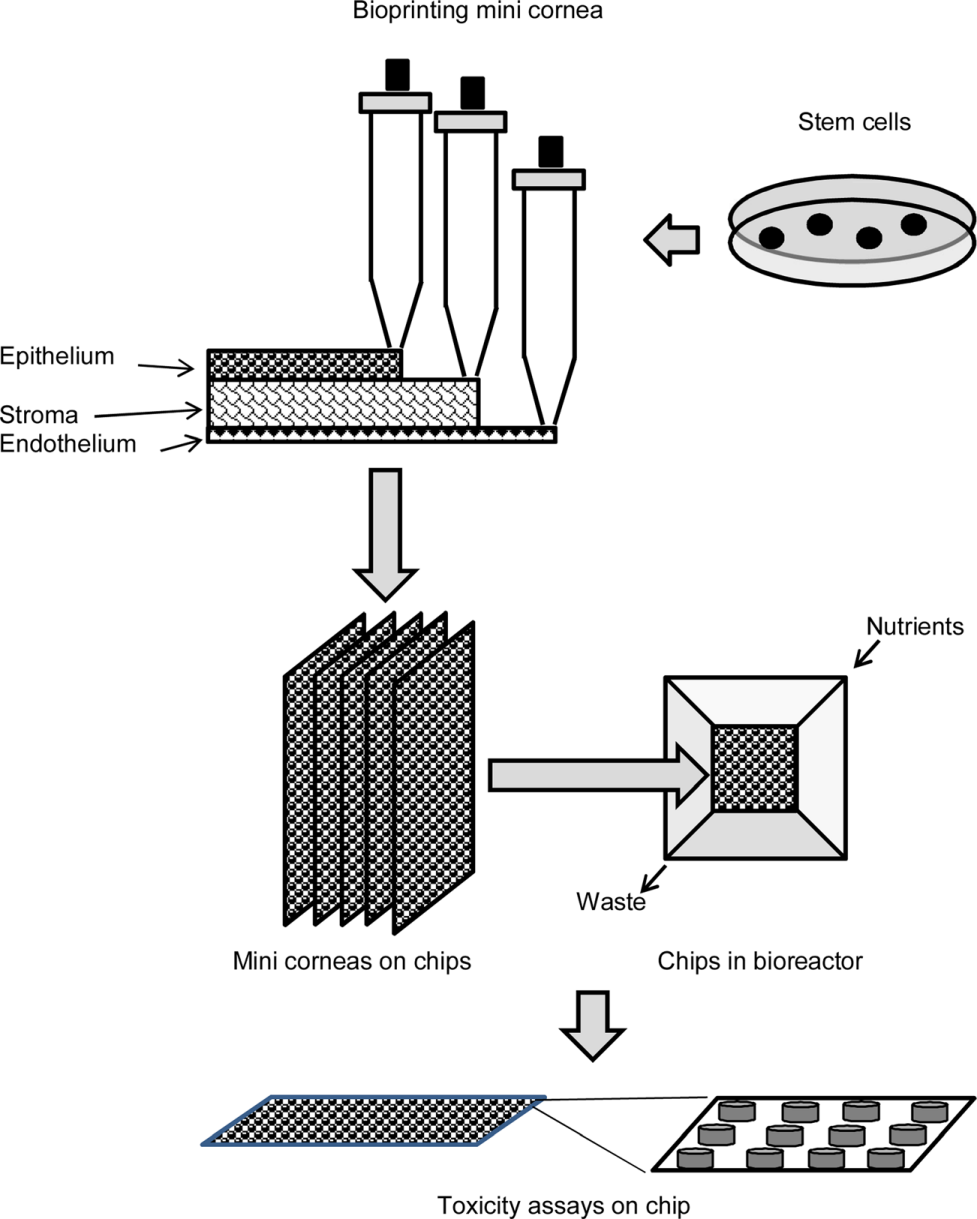
Bioprinting and maturation of miniature corneas

Human embryonic stem cells and induced pluripotent stem cells (iPSC) represent another potential cell source. Under defined conditions, these cells can proliferate indefinitely and maintain their pluripotent phenotype thereby providing potential to generate any cell type [89]. Increasing the availability of human embryonic stem cell lines and iPSC lines may provide a basis of new type of organotypic corneal models for drug toxicity testing. These developments together with the advances in technologies (e.g., bioprinting) offer opportunities to generate 3D organotypic corneal cell cultures [90]. The bioprinted and miniaturized corneas could become tools for ocular toxicity testing (Fig. 4). Currently, only relatively simple and thin structures of living cells and extracellular matrix can be printed in 3D format.

Bioprinting 3D is still a relatively new technology that needs further development. Challenges include optimization of printing process and control of the differentiation of stem cells in 3D culture. It is necessary to understand the mechanisms of cellular damage and behavior in printed formats [90, 91]. Printable liquid biological materials are needed to enable controlled cell differentiation [92]. In principle, combination of modern tissue engineering and stem cell technology could enable successful corneal construction for drug and chemical testing.

However, rigorous testing is needed to validate such models as predictors of ocular toxicity. Currently, the toxicological value of the engineered tissue or bioprinted cell models of the cornea is not known.

## Optimization of the differentiation of corneal epithelial cells

### Culturing process

Morphology and functionality of the corneal cell culture model should be comparable to the normal cornea. The culture conditions should mimic the conditions in vivo in terms of pH, osmolality, temperature, oxygen levels, CO_2_ concentration, and nutrition [8]. Permeable support and co-culture systems, an air-liquid interface, and culture medium components are the main parameters that are used to control differentiation of corneal epithelial cells (Table 1).

**Table 1:** Important components and their function in differentiation of corneal epithelial cells in culture models

Component	Function	References
Permeable support systems		
Laminin, collagen (type I), fibronectin	Promoting differentiation and cell attachment	[4, 35, 36]
Amniotic membrane	Attachment and differentiation of cells	[93]
Insert filters	Polarization of the cells, cells can be fed from the basolateral side	[4, 8]
Coculture systems		
Fibroblasts	Feeder layer, provoking differentiation of cells	[94]
Air-liquid interface	Inducing differentiation	[4, 95]
Supplements		
Ca^2+^, ascorbic acid, hydrocortisone, and retinoic acid	Strengthening barrier function in serum free medium	[96]
Isoproterenol, cholera toxin	Enhancing cell proliferation	[97]
Dimethyl sulfoxide	Differentiation agent	[98]
Epidermal growth factor	Increasing adhesion, proliferation, and spreading	[99]
Insulin, insulin growth factors 1 and 2	Promoting proliferation, inhibiting apoptosis	[100]
Selenium	Preventing oxidative stress	[101]
Transforming growth factor-α	Stimulating cell migration	[102]

Corneal epithelial cells have been cultured in serum-containing media with growth factors, agents promoting cellular proliferation and differentiation, cell attachment factors, and nutrients. However, serum may disturb proliferation and differentiation of corneal epithelial cells [103], since it contains unknown growth inhibitors and activators. Disturbed differentiation might lead to a sub-optimal cell model that does not mimic the normal corneal epithelium leading to a risk of decreased reliability of toxicological predictions. Therefore, defined serum-free culture conditions have been developed [41, 104, 105]. In these conditions, Ca^2+^, ascorbic acid, hydrocortisone, retinoic acid, and transforming growth factor-α are crucial ingredients to support the differentiation of corneal epithelial cells (Table 2). However, optimal culture conditions should be established individually for each cell line. There are also commercial serum-free-defined culture media for epithelial cell cultures (Epilife®, Life Technologies).

**Table 2:** Markers used in evaluation of corneal epithelial-specific differentiation in corneal cell models

Determinants of differentiation	Markers	References
Cellular morphology	Cobblestone morphology, multilayered well-stratified epithelium, microvilli tight junctions, desmosomes	[4, 39]
Basement membrane components	Collagen α5(IV), laminin-1, laminin-5, fibronectin, type VII collagen	[108]
Cytokines	Interleukins IL-lα, IL-lβ, IL-6, IL-8, tumor necrosis factor α	[40]
Growth factors	Transforming growth factors α, β1 and β2, epidermal growth factor, platelet-derived growth factor	[40]
Keratins	Keratin 3 (K3, 64-kDa)	[67]
	Keratin 12 (K12, 55-kDa)	
Metabolic enzymes	Cytochrome P450, glutathione transferase, *N*-acetyltransferase, sulfotransferase	[109]
Transcription factors	Pax6, FoxC1	[110, 111]
Tight junction proteins	Claudins, occludin, ZO-1, ZO-2	[112–114]

### Differentiation markers for corneal epithelium

Differentiation of corneal epithelial cells is often monitored with the expression of markers that have been derived from the primary corneal epithelial cells (Table 2). The markers are important in the assessment of the cell differentiation, but they are not necessarily useful as toxicological marker. However, the epithelial barrier integrity can be a useful end-point, because loosening of the barrier may lead to in vivo exposure of the sensitive corneal endothelium to toxic chemicals. The barrier integrity of corneal epithelial cultures is verified using measurement of transepithelial electrical resistance (TEER) and permeability of paracellular permeants (e.g., mannitol, 6-carboxyfluorescein) [4, 18, 38, 47]. TEER is an indicator for ionic (Na^+^ and Cl^−^) permeability of the intercellular tight junctions [106]. Corneal epithelial cells are considered to have proper tight junctions when TEER recordings are at least 400 Ω cm^2^ [8], but in general it is better to use TEER and paracellular permeability experiments in combination to characterize the barrier of corneal cell models [4]. In addition, molecular weight markers have been used to estimate paracellular porosity and average pore size of isolated cornea and corneal epithelium [47]. This approach gives more complete view on the corneal epithelial barrier. However, universal characterization of the cultured corneal epithelium with transcriptome analysis revealed substantial differences between the culture model and normal human corneal epithelium [107].

## Toxicity tests with corneal cell culture models

Corneal cell culture models are developed to reduce, refine, and replace animal testing. At the moment, the cell culture models do not replace all animal tests, but they are useful in the reduction and refinement of animal experiments. The following sections inform about ocular toxic reactions and the role of the cell culture test systems as predictive models.

### Toxic reactions

Chemical exposure may cause eye irritation that is a painful reaction involving direct action on the pain receptors [115]. Irritation may result in the damage of corneal tissue [116]. The extent of corneal injury depends on the toxicity of the chemical. Typical irritating agents are cationic, anionic and non-ionic surfactants, aldehydes, acids, alcohols, and alkaline substances. Slight irritants injure corneal epithelium, mild and moderate irritants injure corneal epithelium and superficial stroma, and severe irritants may cause damage in all corneal layers. Typical corneal responses to irritation include inflammation, activation, and migration of keratocytes, fibrosis, and neovascularization [117]. Toxic effects in vascularized conjunctival tissue include redness, swelling (chemosis), and discharge [10].

Drugs and metabolites may also induce toxic responses that are not related to direct irritation. Drug-induced toxicity is a complex phenomenon that reflects interactions with target and off-target molecules (e.g., proteins, RNA, and DNA) and alterations of metabolic and signal transduction pathways that may lead to adverse effects [118, 119]. There are huge numbers of potential alterations that may take place, and determination of the molecular mechanisms of toxic actions is a challenging task.

### Toxicity assays and end-points

The principle of toxicity assays in vitro is to expose cells to various drug concentrations over a certain time. The cells are monitored after the incubation and compared to the normal state.

**Fig. 5:**
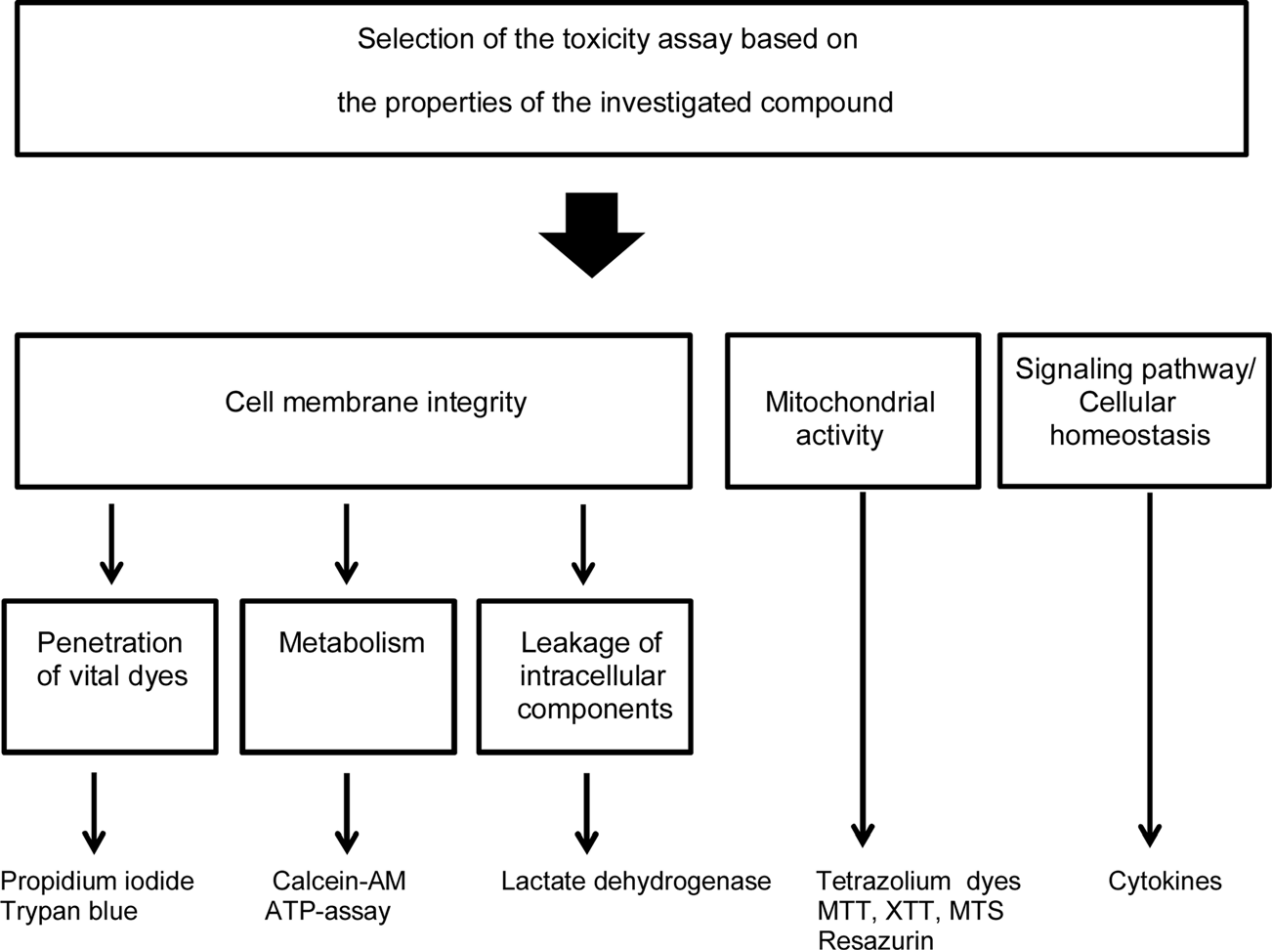
Categories of cellular toxicity assays and their end-points

Cell viability is the most straightforward end-point in toxicity assays. Chemicals may cause cellular injury that induces cytotoxicity via apoptotic and/or necrotic pathways. Common viability assays are based on the integrity of cellular membrane or metabolic activity of the cells. These factors are analyzed using colorimetric-, fluorometric-, or luminescence-based methods [120, 121]. Typically, the rank order of assay sensitivity follows the pattern: luminescence > fluorescence > colorimetry [121–123]. The measurement of the cell membrane integrity is a common method assessing cellular cytotoxicity. Propidium iodide, Trypan blue, calcein-AM, and lactate dehydrogenase are commonly used biomarkers for cell membrane integrity [120, 121, 124] (Fig. 5). Propidium iodide and Trypan blue are unable to cross plasma membrane of viable cells, but permeation can take place through damaged cell membranes resulting in strong nuclear staining of the cells. The lipophilic ester, calcein-AM, permeates into the cells where esterases convert it to fluorescent calcein that is entrapped within the viable cells [124]. Conversion and entrapment do not take place in non-viable cells with leaking plasma membrane. Another option is to determine leakage of intracellular components through the plasma membrane [120, 121]. Lactate dehydrogenase can leak out from the cells only if the plasma membrane has been damaged and its activity in the cell culture medium can be conveniently measured with colorimetric, fluorometric, or luminescence assays (Fig. 5).

Cytokines are often used as biomarkers of cytotoxicity [125]. These signaling molecules have a wide variety of cellular functions, and they are stimulated when cellular homeostasis is altered. Toxic chemicals can induce signaling pathways involved in cytokine release, and cytokine levels can be measured by bioassays or immunoassays (Fig. 5).

Toxic compounds may cause effects on the cellular metabolic activities and, therefore, such changes are useful indicators of cellular toxicity [121]. One of the indicators is ATP metabolism, because the loss of membrane integrity leads to rapidly decreasing levels of ATP that can be detected with fluorescence or luminescence assays. Other widely used metabolic indicators report the activity of cellular oxidation-reduction systems (e.g., MTT, XTT, MTS, resazurin, WST-1) [120, 121, 126]. Unlike dead cells, viable cells reduce the dyes to colored products [124]. The amounts of the colored products are directly proportional to the number of viable cells. Toxic processes, like biological cellular pathways in general, are complicated and highly interactive. Therefore, toxicity assay methods are shifting from single targets to multiplex formats. The multiplex assays can detect multiple biomarkers (viability, cytotoxicity, apoptosis) simultaneously in the same sample [121]. Therefore, more information can be gained from the pathways of necrosis, apoptosis, oxidative stress, and growth arrest. At present, there are no reports on the use of multiplex formats in corneal toxicity assessment. Many single parameter assays have been used in the human corneal toxicity studies (e.g., MTT, propidium iodide, calcein-AM) [22].

ABC efflux transporters are an important part of the cellular protection against toxic agents. They also limit distribution of many clinical drugs into cells [127]. Interestingly, substrates and inhibitors of ABC transporters may interfere with the MTT assay, because the dye is also a substrate to such transporters [128]. Consequently, exposure to the substrates and/or inhibitors of ABC efflux proteins may increase the apparent viability of cells in the MTT assay thereby underestimating the cellular toxicity. Furthermore, other physical or chemical characteristics such as the color of the drug or the ability to reduce used substrate can interfere with toxicity experiments and thus give incorrect results [129].

Overall, interpretation of toxicity assay results can be complicated. Toxicity can result from various cellular events, such as changes in enzymatic activity, differentiation, proliferation, morphology, cell functions, and cell detachment [130, 131]. Insights into the biochemical mechanisms may be obtained through systems approaches (proteomics, transcriptomics, metabolomics) that are emerging tools in predictive toxicology [118, 132]. Furthermore, the data obtained from “omics” technologies can be combined with structure-based information on toxicity from computational in silico models, resulting in so-called hybrid toxicity modeling systems [133]. Structure-based predictive toxicity models could be based on the fingerprints, archetypical changes in the biochemical pathways that are caused by a certain chemical class leading to defined toxic symptoms. Such information would be very useful, but it requires plenty of experimental work before such models can be established for corneal toxicity. So far, such models have not been utilized to assess the corneal toxicity. Traditional simple end-point assays do not yield mechanistic insights, like systems biology, but they are attractive due to their simplicity. At the moment, the “omics” techniques are far too expensive, slow, and resource intensive for the practical assessment of corneal toxicity.

### Toxicity tests with monolayer cultures of human corneal epithelial cells

A monolayer culture assay with primary or immortalized cells grown on plastic wells is the simplest corneal cell toxicity model. Human primary corneal epithelial [19, 21], and immortalized corneal epithelial cells [19, 22, 134, 135] have been used to evaluate toxicity of natural tear substitutes, various ophthalmic drugs, and pharmaceutical excipients. It is expected that a simple culture of non-differentiated corneal cells should predict the toxic reactions in the intact cornea in vivo. Saarinen-Savolainen et al. [22] showed correlation between cellular toxicity in the corneal epithelial cell line and the corneal toxicity in vivo. This report also paid attention to the concentrations of in vitro and in vivo exposure.

Only a few studies have compared the toxicity of ophthalmic drugs in corneal and non-corneal cells. Cheong and coworkers [19] tested the susceptibility of primary and immortalized corneal and retinal cells, human skin keratinocytes, and fibroblasts to eight β-blockers (propranolol, alprenolol, atenolol, labetalol, metoprolol, pindolol, timolol, bisoprolol). The standard MTT assay did not reveal any differences in the toxicity of β-blockers in keratinocytes, fibroblasts, and human corneal epithelial cell lines.

Monolayers of human primary or immortalized corneal epithelial cell cultures do not adequately represent the whole cornea with extensive cell-cell and cell-matrix interactions. These factors can have a marked influence on the toxicity of substances. Furthermore, there is a great inter-laboratory variation in the toxicity data from corneal cell monolayer studies [136]. An increasing number of researchers have questioned the validity of studying toxicity in monolayer cell cultures, and therefore, there has been a growing tendency towards more complex and sophisticated cell models.

### Toxicity tests with human corneal endothelial cells

Human corneal endothelial cells have been used to evaluate toxicity of ophthalmic solutions, drugs, and cleaning procedures in ophthalmic surgery [137–140]. Human corneal endothelial cells are the best choice for this kind of in vitro safety studies for intra-cameral agents. Anti-glaucoma drug formulations with preservatives were more toxic than preservative-free formulations. However, dilution of these formulations up to 100 times diminished the cytotoxicity substantially suggesting that the endothelial toxicity risk in vivo is small. Toxicity results of ophthalmic solutions suggest that the survival rates of primary corneal endothelial cells are in general comparable to those of bovine and rabbit corneal epithelial and SIRC cell lines, but the rank-order is compound dependent [137, 141].

### Toxicity tests with three-dimensional cell culture models

There is a growing interest to use corneal epithelial models in which the cells are cultured on a synthetic micro-porous membrane at air-liquid interface. This results in a multi-layered epithelium with tight junctions on the apical side [4]. Usually, these models have been developed for in vitro drug permeation studies, but it is possible to use them also for toxicity assessment. The European Union Reference Laboratory for alternatives to animal testing (EURL ECVAM) has not fully validated any corneal cell model for eye irritation tests yet.

The commercially available EpiOcular cell culture system by MatTek has been used in toxicity and irritancy tests. It consists of human skin-derived epidermal keratinocytes that have been cultured to form stratified squamous epithelium [23]. Although EpiOcular is based on non-corneal cells, it has relatively good ability to find ocular irritants, non-irritants, and to discriminate them from each other. The model was tested with 105 different chemicals, and EURL ECVAM concluded that the model was predictive, even though the agency had some reservations [142]. According to ESAC, EpiOcular™ EIT had satisfied the requirements regarding transferability, reproducibility, and predictive capacity in the assessment of irritation potential of chemicals. ESAC also pointed out that confidence in the test method would be increased if supplementary investigations confirm that the method correctly classifies a representative sample of products from different sectorial classes as defined in REACH. It is unlikely that this model would be useful in more subtle forms of toxic reactions (e.g., inflammatory responses), because epidermis and corneal epithelium are quite different tissues.

Another commercially available reconstructed human corneal epithelial model (SkinEthic HCE model) was used to test 435 substances from the cosmetic industrial domain including surfactants, polymers, fatty derivatives, silicons, dyes, solvents, natural extracts, and preservatives [20]. Later, transferability, reproducibility, and predictive capacity of this model were investigated in studies performed in three laboratories using both liquids and solids [129, 143]. The overall accuracy of the model was 83.7 % (200 chemicals), specificity 72.1 % (103 chemicals), and sensitivity of 95.2 % (97 chemicals) [143]. However, EURL ECVAM Status Report concluded that SkinEthic™ HCE would require optimization and further validation [142].

Recently, another commercial cell model (LabCyte CORNEA-MODEL; Japan Tissue Engineering Co., Ltd.) has been tested [144]. In this model, differentiated human corneal epithelium was obtained on filter substrate. Sixty-one chemicals were tested, and the model appeared to be a relevant and reliable predictor of in vivo toxicity.

Jung et al. [145] reconstructed human corneal epithelial model (MCTT HCE model) from primary human limbal epithelial cells that were obtained from human subjects undergoing corneal transplantation. The MCTT HCE model develops morphology and biomarkers similar to intact human corneal epithelium within 7 days of cultivation on a polycarbonate filter. This in vitro human corneal epithelial model showed high predictive ability of irritant and non-irritant compounds. Advanced 3D human corneal model that includes stromal matrix with incorporated immortalized keratocytes and with immortalized human corneal epithelial cells (HCE) was recently reported [146]. This model is an intermediate step between a corneal epithelial model and full 3D corneal equivalent. This model was tested using 20 chemicals with different eye-irritating potential in two independent laboratories. The model showed predictive capability, but the levels were less than those with simpler models.

In principle, complex 3D tissue models should mimic human cornea better than simple models. So far, the difference is not obvious. In addition to the macroscopic appearance of the models, they should mimic also the cellular biological networks in the corneal cells. This aspect has not been studied in detail, but one example showed substantial difference in the systems biology of a corneal epithelial cell model compared to normal human ex vivo corneal epithelium [107].

### Similarity between the cell models and human cornea

HCE-T cells (simian virus SV-40-immortalized human corneal epithelial cells) have been used widely in ophthalmology, since they were published for the first time [38, 39]. However, array-based comparative genomic hybridization analysis demonstrated that genomic content of these cells is altered, and heterogeneous cell populations exist in monolayer cultures [43]. Furthermore, high-density oligonucleotide microarray analysis revealed significant differences between the gene expression profiles of the 3D HCE-T model and freshly isolated human corneal epithelium [107]. HCE-T cells form a stratified, compact structure that resembles in many ways native human corneal epithelium (tight barrier with TEER value >300 Ω cm^2^, desmosomes, tight junctions, apical microvilli), but still 22 % of the genes were over-expressed and 14 % under-expressed. It has been shown earlier that cytokeratin expression of immortalized human corneal epithelial cells on filters does not resemble native cornea [147]. We should also note that the same cell line may show different gene expression patterns in distinct laboratories [136]. This aspect of SV-40 immortalized cells may also influence the behavior of the hemi-cornea model [146].

As described earlier in this review, some human corneal culture models, such as EpiOcular [23], SkinEthic HCE model [20, 148], LabCyte CORNEA-MODEL [144], and MCTT HCE model [145], have been used in in vitro irritation studies with rather good correlations to in vivo irritation. EpiOcular contains human skin-derived epidermal keratinocytes, and no corneal cells at all [23]. Therefore, it is unlikely that this would be an optimal model for ocular toxicity testing, whereas the LabCyte CORNEA-MODEL is based on normal human corneal epithelial cells [144], and the MCTT HCE model was constructed using human limbal epithelial cells [145]. Thus, these models may be closer to human cornea than EpiOcular and SkinEthic HCE models. Anyway, no commercial model has been characterized at systems biology level for gene expression.

Direct corneal irritation after topical administration is a strong acute effect, and, therefore, it is not surprising that toxicity of the irritant compounds becomes evident even in non-corneal cells [23] or monolayer cultures of corneal epithelial cell lines [22]. However, side effects of ocular drug candidates are milder, and they may show distinct mechanisms and long-term effects. Studies of these phenomena require cell models that are closely mimicking the human cornea. Previously, we demonstrated that commercial epidermal cell models with poorly developed barrier properties showed poor correlation with in vivo skin irritation, whereas a skin model with decent barrier predicted in vivo adverse effects much more reliably [149, 150].

Drug exposure time is a crucial difference between the cell culture experiments and the in vivo situation after topical administration. After topical ocular administration, the drug concentration in the tear fluid drops rapidly within a few minutes, but the drug may be applied continuously using multiple dosing regimen [1]. Drug toxicity is a function of exposure (concentration, dose), and the concentrations in the pre-corneal fluid are changing rapidly. The concentration range can be estimated based on the literature or simulated [151]. It is not well understood how this aspect influences the reliability of toxicological predictions, but several drug concentrations and exposure times should be used in vitro to mimic the exposure conditions in vivo [121].

## Conclusions

Various cell models with different complexity have been used in the past. Some organotypic 3D models have been thoroughly evaluated, but not yet validated for toxicity predictions. Some simple models seem to be useful for toxicity screening, and their advantages include speed and low cost. More complex 3D models have some specific advantages, such as monitoring of the potential toxic effects in non-epithelial cell types (endothelial cells, keratocytes) and effects on the structure of the tissues (e.g., erosion effects).

The mechanisms of corneal toxicity are not well understood, and this makes translation of the results to the human eye in vivo difficult. Currently, the predictive capacity of the cell models is usually evaluated using the Draize test as gold standard, but the relevance of the Draize test as a predictor of human corneal toxicity is not clear either.

One of the challenges for the future will be to further improve human corneal culture models in such a way that they resemble native human cornea as closely as possible. Integration of bioprinting with modern tissue engineering may provide such progress, but this field is at an early stage and no clear guidelines on the best practices are available. More effort is needed toward better characterization of constructed human corneal culture models using multiplexed or systems biology level approaches.
